# Is It Right

**Published:** 1857-05

**Authors:** 


### IS IT RIGHT

FOR a religious newspaper to put under the heading of "*general news*" the following? It might be useful to inquire, was the article paid for? When religious newspapers go down to the trickeries of the secular press, and practise the same business morals, we may reasonably expect a decline in their religious influence. The publisher of the paper, and the editor too, know very well that this article has been travelling the rounds of the press for months, and all endorse the statement that it is an item of "news." Insidious untruth is everywhere dangerous, but coming from Christian men, from clerical editors, we may well inquire, where are we going?

The whole design of the article is to spread the name of secret medicines, and religious newspapers lend their aid in that direction. We simply inquire, "*Is it right?*"

Dr. * * *, in his travels on the Cape of Good Hope, says: I found very frequently among the Dutch Boors of the back country, * * *, which they keep hung up by a thong around the neck of the bottle to a peg over their hammocks. Indeed this seems to be their sole protection against the throat and lung disorders which are quite prevalent among them. I thought it a speaking comment on the practical genius of the American people, that they should furnish the staple, I believe the only remedy this people buy to use. Asking if they used the same manufacturer's pills, they told me that better purgatives grew all around them than anybody could prepare.

